# Traumatic experiences and place of occurrence: An analysis of sex differences among a sample of recently arrived immigrant adults from Latin America

**DOI:** 10.1371/journal.pone.0302363

**Published:** 2024-06-14

**Authors:** Laura X. Vargas, Mary D. Sammel, Therese S. Richmond, Connie M. Ulrich, Zachary D. Giano, Lily Berkowitz, C. Neill Epperson

**Affiliations:** 1 Department of Psychiatry, School of Medicine, University of Colorado-Anschutz Medical Campus, Aurora, CO, United States of America; 2 Department of Biostatistics and Informatics, Colorado School of Public Health, University of Colorado-Anschutz Medical Campus, Aurora, CO, United States of America; 3 Department of Behavioral Health Sciences, University of Pennsylvania School of Nursing, Philadelphia, PA, United States of America; 4 Center for Innovative Design & Analysis, Colorado School of Public Health, University of Colorado Anschutz Medical Campus, Aurora, CO, United States of America; Universidade do Estado do Rio de Janeiro, BRAZIL

## Abstract

With increasing violence, political, and economic instability in Latin America, there is a record number of migrants crossing the U.S. southern border. Latin American migrants are often exposed to traumatic events before leaving their home country and during migration. While prior studies document that sex may play a role in types of traumatic exposure, few studies compare differences in traumatic exposure by sex and place of occurrence of recently arrived immigrants. Addressing this gap, we recruited 120 adults who had recently crossed the U.S.-Mexico border. Participants completed questionnaires to characterize trauma exposures in their home country and during their migration journey. Results found that men reported higher levels of exposure to combat situations, while women were more likely to experience sexual assault. Both combat exposure and sexual traumas occurred more often in home countries than during migration. More than half of the full sample reported being threatened with a firearm. These data confirm gender differences in type of trauma and that exposures in the country of origin may provide the impetus to migrate.

## Introduction

Historically, migration from Latin America to the U.S. predominantly consisted of single, working-age males seeking economic opportunity [1]. Latin American migrants from four countries (i.e., El Salvador, Guatemala, Honduras, and Mexico) account for over 90% of all apprehensions by United States (U.S.) Customs and Border Patrol (CBP) at the southern border, but the proportion of migrants from other origin countries in Latin America has increased in recent years. Rising violence in the region contributes to changes in the demographic composition of migrants. Given the demographic transition in the Latinx migrant population to the U.S. and the rise in forced migration from Latin America, it is not surprising that limited prior research has examined sex differences in trauma exposure among recently arrived Latinx immigrants. These changes in demographic patterns offer the opportunity to study immigrants from a wider array of countries of origin. The purpose of this research is to fill a gap in understanding how traumatic experiences of recently arrived immigrant adults from Latin America may vary by sex and by place of occurrence (i.e., in the home country, during the migration journey, or both) prior to their arrival to the U.S.

The U.S. Latinx population has increased nearly nine-fold since 1960 and is projected to grow to 107 million by 2065 [2]. Exposure to violence in Latin America is a significant contributor to migration of adults and families to the U.S. [3, 4]. Latin America accounts for 36% of the world’s homicides despite representing only 8% of the world’s population [5]. Migration as a result of forced displacement is rising [6] and contributes to the increasing diversity of migrants (i.e. more women, children, older adults). Several studies in various Latin American countries report a wide exposure to potentially traumatic events [7–13].

Many studies of traumatic exposure conducted in Latin American countries either report no overall association between prior traumatic exposure (PTE) and sex or that males are more likely to report a PTE [7, 9–11, 14, 15]. These studies also suggests that sex differences are dependent on the type of event; for example, most studies find that females are significantly more likely to report experiencing sexual violence [7, 9–11, 15]. Thus, as violence is a widespread problem throughout the Latin American region, it is important to examine trauma exposure differences by sex among newly arrived immigrants from Latin America.

While these studies illustrate that people migrating from Latin America may experience potentially traumatic events in their home countries, they do not specifically refer to the experiences of migrants. Studies highlight that the relationship of migration and mental health [16] is mediated by trauma exposure, and various other covariates including sex [12, 17–19]. Very few studies focus on in depth research of specific trauma experiences of women immigrants [19]. Some studies have focused on the impacts of exposure to trauma and mental health among Latinx immigrants [11, 20–22], though few have studied exposure to trauma among recent Latinx immigrants [23, 24]. The rationale for our study is that we present a first time comparison of traumatic experiences of recent immigrant adults from Latin America by sex and place of occurrence.

## Methods

### Design and setting

This cross-sectional descriptive study was reviewed and approved by the Colorado Multiple Institutional Review Board (COMIRB), the Institutional Review Board (IRB) for the University of Colorado Anschutz Medical Campus. The setting for this study was a humanitarian respite center located on the U.S. side of the U.S.-Mexico border.

### Participants

Adults ages 18+, Spanish-speaking, who arrived in the U.S. within the past 14 days of the date of interview, were from a Latin American country, and able to give informed consent were recruited to participate in the study. In the rare case that potential participants only spoke English were eligible for participation; potential participants who only spoke a non-Spanish indigenous or regional dialect and did not understand or speak Spanish or English were excluded.

### Data collection

We approached 147 individuals for recruitment after having their basic needs met and 120 were enrolled and surveyed if they had at least two hours to wait for transportation at a humanitarian respite center in McAllen, Texas. Study recruitment took place from April 2022 through March 2023. The study purpose and procedures were explained, all questions answered, and verbal informed consent obtained. Participants were interviewed in a private area to ensure confidentiality. To thank participants for their time, we provided a $50 gift card at survey completion to a major retail store in the U.S. Data were stored according to a unique code given to each participant in lieu of participant names. Some of the reasons provided by those approached for recruitment but who did not enroll in the study were: they did not have sufficient time, they were tired from their journey, or they preferred not to take part in the study.

### Measures

Exposure to trauma. Trauma exposure is defined as exposure to violence, crime, sexual exploitation, deprivation, discrimination, or other intentional and unintentional traumatic experiences that occur before and during migration to the U.S. A combination of measures were used to capture a history of potentially traumatic experiences: (1) an adapted version in Spanish of the validated Harvard Trauma Questionnaire (HTQ) [25–27] Peruvian version that measures (a) material deprivation, (b) war-like conditions, (c) bodily injury, (d) forced confinement/coercion, (e) disappearance/death/injury of loved ones, (f) witnessing violence to others, and (g) kidnapping and extortion; (2) exposure to discrimination from the National Latino and Asian American Study (NLAAS) included items assessing everyday experiences of discrimination and perceived discrimination that show high internal consistency both in Spanish and English [28]; and (3) a multiple choice question asking immigrants about their reasons for migrating, with the following answers: settlement (long term/permanent stay); employment; education or training; marriage, family reunification, or family formation; forced displacement (refugees, asylum seekers, temporary protection, etc.); displacement due to climate events; or other reason: where participants were asked to elaborate on that reason.

Demographic information. Individual demographic characteristics from the World Health Organization World Mental Health Composite International Diagnostic Interview (WHO WMH-CIDI) [29] include sex (male/female), age, marital status, education, main language spoken at home, and employment in the previous year (i.e. the question asks “how many months did you work in previous 12 months?”). We complemented CIDI demographics with questions about country of origin, the migration journey, such as time since leaving home country, prior migration experience, illnesses, and access to food, water, and medical care. We also ask two questions to understand participants English language abilities, asking them to self-rate (on a scale of 1–10 with 1 being “not at all” and 10 being “perfectly”) their ability to understand spoken English and speak English, respectively.

### Statistical analysis

**Fig 1:**
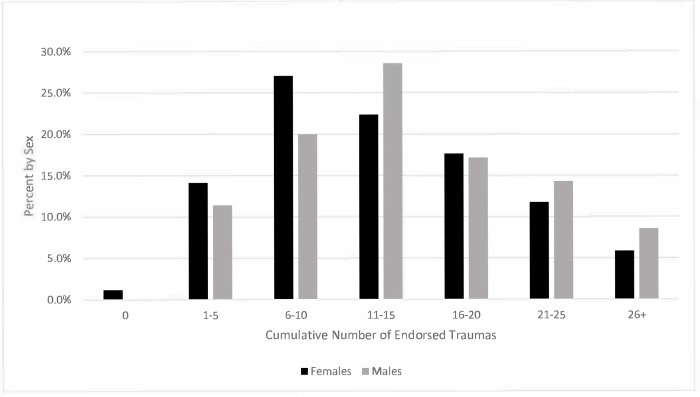
Cumulative traumas by sex.

Frequency and descriptive statistics were generated with significance testing between males and females using Pearson Chi-square and t-tests where appropriate. Frequencies were calculated for the 45 trauma items for the full sample, males, and females; including the location of the trauma, when applicable. Two sets of testing were then conducted: the first evaluated the association between sex (male/female) and trauma type (yes/no) using Chi-square tests. A second Chi-square test was conducted to assess the association between sex (male/female) and a 4-level variable regarding the location/timing of trauma (no exposure/home country/during migration/both home country and migration). Due to small cell sizes, a number of comparisons used Fisher’s exact test (noted in Table 3). Finally, we compared cumulative count of the number of trauma experiences by sex and graphed them by different occurrence categories (Fig 1. Cumulative Traumas by Sex).

Migration journey duration was calculated in days by subtracting departure date from interview date. The 15^th^ day of the month was assigned to those who only recalled month and year of departure if departure took place prior to 1/1/2020. Three participants were missing the day of departure for a date after 1/1/2020 or had an otherwise unusable date (e.g., missing month or year), their journey length was considered missing and was excluded only from journey length analysis.

## Results

### Participant characteristics

The convenience sample consisted mostly of 120 asylum-seeking adults who were in transit to a U.S. destination. Although undocumented migrants may come to the U.S., in the current environment and with existing policies, most people at the study enrollment site are asylum-seeking immigrants.

Table 1 presents demographic and migration journey characteristics. Nearly 70% of the sample were female participants with an average age of 33.9 years. Male participants were significantly older (36.5) than female participants (32.8) (p = 0.020). Nearly 90% of respondents were traveling with children and the average number of children per respondent was 2.2. Male participants worked on average nearly two months more than female participants in the past year (p = 0.041). While Spanish was the predominant language spoken at home among participants, males self-rated their ability to speak and understand English as higher compared to females. Nearly 90% of the sample responded that this was their first time in the U.S., but men were more likely than women to endorse having been to the U.S. previously. Just over 80% was from Central America, with 10% from South America and nearly 7% were from the Caribbean. Additionally, 15.6% of the total sample were pregnant women or men who were travelling with a pregnant partner, and 94.4% of the sample were traveling with children.

**Table 1:** Sample demographics (N = 120).

			Range, if Applicable	Full Sample	Females	Males	p-value	
				*%/M (StDev)*	*%/M*	*%/M*		
*Demographics*								
	Female			69.7	-	-		
	Married			39.3	32.9	55.6		
	Live w/Significant Other			67.2	58.8	88.9		
	Pregnant/Spouse is Pregnant			15.6	18.8	8.3		
	Age			*33*.*9 (8*.*2)*	*32*.*8 (7*.*9)*	*36*.*5 (8*.*5)*	**.020**	a
	Number of Children			*2*.*17 (1*.*3)*	*2*.*08 (1*.*2)*	*2*.*3 (1*.*4)*	.275	a
	Months Employed in Past Year			*6*.*91 (4*.*9)*	*6*.*3 (5*.*1)*	*8*.*31 (4*.*1)*	**.041**	a
	Years of Education						.141	a
		0		2.5	1.2	2.0		
		1–6		27.4	27.1	27.8		
		7–9		20.6	21.2	19.5		
		10–12		24.8	22.4	30.6		
		13–15		14.1	14.1	13.9		
		16+		10.6	14	2.8		
*Language Abilities*								
	Spanish Spoken at Home			93.4	95.3	91.7	.423	b
	Ability to Understand English		1–10	*3*.*2 (2*.*3)*	*2*.*8 (2*.*1)*	*3*.*8 (2*.*7)*	**.023**	a
	Ability to Speak English		1–10	*2*.*6 (2*.*1)*	*2*.*3 (1*.*6)*	*3*.*3 (2*.*9)*	**.013**	a
*Immigration Characteristics*								
	First time in U.S.			90.9	92.9	86.1	.236	b
	First time Crossing U.S. Border			80.2	82.4	75.0	.358	b
	Traveling with Children			89.3	87.1	94.4	.234	b
	No food for more than a day			72.7	72.9	72.2	.936	b
	Dehydration			62.8	58.8	72.2	.166	b
	Illness/Injury			58.7	60.0	55.6	.653	b
	Lack of Medical Care			68.6	68.2	69.4	.897	b
	Mental Health Crisis			75.2	77.6	69.4	.344	b
	Lack of Personal Hygiene			65.3	65.9	63.9	.835	b
	Lack of Food for Children			63.3	61.2	68.6	.449	b
	Apprehended Upon Arrival			95.0	96.5	91.7	.270	b
	Days Apprehended (if applicable)			2.0	1.9	2.1	.105	a
	Days from start of journey to interview			131.2	104.1	200.1		
Country/Region of Origin								
	Mexico			1.7	2.4	-		
	Central America			80.6	81.0	80.5		
	Caribbean			6.8	5.9	8.3		
	South America			10.9	10.7	13.2		

Note: a = t-test, b = Chi Square test

All but 5% were apprehended upon arrival at the U.S. southern border and endorsed average time in apprehension of just over 2 days. Conditions of the migration journey showed that just over 72% experienced a lack of food for more than a day and over 62% experienced a lack of water for more than 24 hours. Nearly 60% experienced an illness or injury during their journey and 68% endorsed a lack health care services when they needed them. Sixty three percent of participants did not have access to food for their children for more than a day and 65% did not have access to adequate personal hygiene conditions and products. Nearly three quarters of participants endorsed experiencing at least one mental health problem during their journey. The average time from the date participants left their home country to the time of interview was 131 days.

### Country demographics and experiences of trauma

Table 2 presents country demographics. Participants came from eleven countries in Latin America and the Caribbean, with the largest portion of the sample from Nicaragua (38.6%). The number of days participants spent migrating (from the date they left their home country, to the date they were interviewed after arriving to the U.S.) ranged widely by country of origin with participants from Haiti migrating on average for over 2,000 days and some participants from countries like Peru and Colombia migrating in under 10 days on average. Most participants from countries other than Peru, Colombia and Ecuador spent a prolonged period of more than one week in Mexico, though a smaller proportion of those participants endorsed having been held or spent time in Mexico by force.

**Table 2:** Country demographics.

					Migration	Migration	% Prolonged	
		Full Sample	Females	Males	Days	Days	Stay in	% Held in
		(%)	(%)	(%)	M/StDev	Median	Mexico	Mexico
*Country of Origin*								
	Colombia	1.7	2.4	-	9.0 (7.1)	9.0	50.0	0.0
	Cuba	2.6	3.5	-	23.0 (4.0)	23.0	100.0	33.3
	Ecuador	2.6	2.4	2.8	13.0 (6.9)	9.0	33.3	0.0
	El Salvador	11.5	12.7	8.3	38.4 (74.7)	15.0	100.0	50.0
	Guatemala	9.1	11.8	2.8	40.4 (94.7)	12.0	90.9	20.0
	Haiti	4.2	2.4	8.3	1795.0 (1030.8)	2042.0	100.0	0.0
	Honduras	21.4	21.2	22.2	98.6 (206.8)	25.0	100.0	50.0
	Mexico	1.7	2.4	-	215.0 (14.1)	215.0	100.0	100.0
	Nicaragua	38.6	35.3	47.2	25.9 (15.9)	20.5	87.0	28.2
	Peru	4.9	3.5	8.4	7.6 (2.8)	9.0	50.0	66.7
	Venezuela	1.7	2.4	-	50.0 (8.4)	50.0	100.0	100.0

Top Reasons for Immigration

Forced displacement (refugees, asylum seekers, temporary protection, etc.); n = 63

Employment; n = 14

Other: Economic and political situation in home country; n = 13

Table 3 presents descriptive statistics of experiences of trauma in the sample measured by the HTQ and four additional trauma exposure questions. The first column of results describes the percent of the sample that experienced each of the specific traumas. Results are ranked by percent of occurrence from high (top of the table) to low. The subsequent columns present the percent of males and females that did not experience each trauma, and if exposed to trauma, at what point in the trajectory it occurred. The final two columns present the chi-square significance in the difference between male and female participants; as well as chi-square and Fisher’s exact test of difference between male and female participants and the place where trauma occurred (or if it did not occur).

**Table 3:** Harvard Trauma Questionnaire results.

	% of Trauma by Full Sample	Males				Females				χ^2^	χ^2^
										(sex & 2-level trauma)	(sex & 4-level place)
		No Exposure	Home Country	After	Both	No Exposure	Home Country	After	Both		
Forced to hide	75.0	19.4	22.2	38.9	19.4	28.2	9.4	40.0	22.4	.148	.261
Lack of food or water	70.0	36.1	5.6	25.0	33.3	28.2	9.4	28.2	34.1	.327	.784
Confined to home because of danger outside	70.0	27.8	0.0	55.6	16.7	31.8	44.7	4.7	18.8	.335	.473
Extortion or robbery	62.5	33.3	13.9	25.0	27.8	40.0	15.3	25.9	18.8	.252	.734
Ill health without access to medical care	59.2	41.7	13.9	11.1	33.3	41.2	12.9	18.8	27.1	.536	.736
Forced evacuation under dangerous conditions	59.2	30.6	55.6	5.6	8.3	45.9	36.5	7.1	10.6	.059	.279
Lack of shelter	53.3	58.3	13.9	11.1	16.7	42.4	24.7	8.2	24.7	.101	.294
Exposure to frequent and unrelenting fun fire	53.3	38.9	47.2	2.8	11.1	50.6	37.6	5.9	5.9	.127	.435
Serious physical injury of family/friend due to combat	52.5	69.4	27.8	0.0	2.8	76.5	22.4	0.0	1.2	.249	.647
Murder, or death due to violence, of other family/friend	52.5	38.9	58.3	2.8	0.0	51.8	48.2	0.0	0.0	.104	.154
Forced separation from family members	51.7	55.6	16.7	25.0	2.8	45.9	25.9	24.7	3.5	.262	.692
Witness beatings to head or body	45.0	47.2	47.2	2.8	2.8	58.8	0.0	37.6	3.5	.134	.309
Beating to the body	40.0	58.3	41.7	0.0	0.0	61.2	32.9	1.2	4.7	.417	.441
Confiscation or destruction of personal property	39.2	69.4	13.9	5.6	11.1	57.6	22.4	10.6	9.4	.182	.519
Disappearance or kidnapping of other family/friend	33.3	69.4	22.2	8.3	0.0	65.9	25.9	8.2	0.0	.476	.912
Enforced isolation from others	30.8	72.2	11.1	11.1	5.6	68.2	10.6	14.1	7.1	.454	.956
Combat situation (e.g. shelling and grenade attacks)	25.8	58.3	41.7	0.0	0.0	81.2	18.8	0.0	0.0	**.007**	**.012** *
Serious physical injury from combat situation	25.8	52.8	44.4	2.8	0.0	45.9	49.4	1.2	3.5	.362	.563
Other types of sexual abuse or sexual humiliation	24.2	91.7	8.3	0.0	0.0	69.4	24.7	4.7	1.2	**.007**	**.043** *
Witness killing/murder	22.5	72.2	25.0	0.0	2.8	80.0	18.8	1.2	0.0	.215	.328
Present while someone searched your home	20.8	77.8	0.0	19.4	2.8	80.0	16.5	2.4	1.2	.451	.709
Kidnapped	19.3	77.8	2.8	16.7	2.8	82.4	2.4	15.3	0.0	.347	.480
Someone was forced to betray you, risking death/injury	19.3	77.8	19.4	2.8	0.0	82.4	0.0	16.5	1.2	.347	.395
Rape	17.5	97.2	2.8	0.0	0.0	76.5	18.8	2.4	2.4	**.004**	**.035** *
Torture (i.e., beating, mutilation, etc)	16.7	80.6	13.9	2.8	2.8	84.7	12.9	2.4	0.0	.352	.485
Forced labor (like animal or slave)	14.2	86.1	11.1	0.0	2.8	85.9	10.6	2.4	1.2	.593	.742
Imprisonment	10.8	83.3	11.1	2.8	2.8	91.8	2.4	5.9	0.0	.136	.053*
Witness torture	10.8	80.6	16.7	0.0	2.8	92.9	4.7	1.2	1.2	**.044**	.088*
Knifing or axing	7.5	86.1	13.9	0.0	0.0	95.3	4.7	0.0	0.0	.081	.124*
Murder, or death due to violence, of spouse	5.8	100.0	0.0	0.0	0.0	91.8	8.2	0.0	0.0	.083	.102
Disappearance or kidnapping of spouse	5.8	94.4	2.8	2.8	0.0	94.1	4.7	1.2	0.0	.668	.733
Witness rape or sexual abuse	5.0	100.0	0.0	0.0	0.0	92.9	3.5	3.5	0.0	.120	.263
Forced to physically harm non-family/friend	3.3	97.2	2.8	0.0	0.0	97.6	2.4	0.0	0.0	.648	.657
Murder, or death due to violence, of son or daughter	3.3	91.7	8.3	0.0	0.0	98.8	0.0	1.2	0.0	.074	**.025** *
Prevented from burying someone	2.6	97.2	2.8	0.0	0.0	97.6	2.4	0.0	0.0	.634	.657
Forced to physically harm family member, or friend	2.5	91.7	8.3	0.0	0.0	98.8	1.2	0.0	0.0	.074	.078*
Forced to destroy someone else’s property or possessions	2.5	94.4	5.6	0.0	0.0	98.8	1.2	0.0	0.0	.203	.211
Forced to betray family/friend risking their death/injury	2.5	97.2	2.8	0.0	0.0	97.6	2.4	0.0	0.0	.648	.657
Forced to betray non-fam./friend risking their death/injury	2.5	94.4	5.6	0.0	0.0	98.8	1.2	0.0	0.0	.203	.211
Disappearance or kidnapping of son or daughter	2.5	100.0	0.0	0.0	0.0	96.5	2.4	1.2	0.0	.352	.521
Forced to find and bury bodies	1.7	97.2	2.8	0.0	0.0	98.8	0.0	1.2	0.0	.500	.248
Other Trauma Questions											
Have you ever witnessed an armed conflict/confrontation?	63.3	27.8	0.0	63.9	8.3	41.2	48.2	3.5	7.1	.081	.294
Have you ever been threatened with a firearm?	50.0	30.6	47.2	11.1	11.1	58.8	32.9	2.4	5.9	**.002**	**.011** *
Have you ever been threatened with any other weapon?	30.8	66.7	0.0	30.6	2.8	70.6	24.7	3.5	1.2	.375	.563
Have you suffered from natural disaster?	45.8	52.8	47.2	0.0	0.0	55.3	44.7	0.0	0.0	.426	.843

* = Fisher’s Exact test p-value

The most endorsed trauma identified was being forced to hide, which was experienced by 75.0% of the sample with the largest percentage of people endorsing that this type of trauma occurred during migration. The places where trauma experiences occurred varied by trauma type, with ten types of traumatic experiences having occurred exclusively in participants’ home country (experiencing a war/combat situation, being knifed/axed, experiencing the murder of a spouse, being forced to physically harm both family/friends and non-family/friends, being forced to betray both family/friends and non-family/friends, being forced to destroy the property of others, and experiencing a natural disaster such a floods, droughts, earthquakes).

A war/combat experience only occurred in participants’ home country, and men were more likely than women to experience this form of trauma (58.3% vs 18.8%; p = 0.007). Female participants experienced significantly more rape (p = 0.004) and other forms of sexual abuse (p = 0.007) than men and this difference persisted when we considered place of occurrence (p = 0.035 and p = 0.043 respectively). Experiencing imprisonment was more likely among men than women in their home country, but more likely among women than men during migration. Men were significantly more likely to witness torture than women (p = 0.044), particularly in their home country or in both contexts (before and after leaving their home country). Men were more likely to experience the murder or death due to violence of a son/daughter in their home country, while women were more likely to experience this trauma during their migration journey (p = 0.025). Finally, half of the total sample endorsed being threatened with a firearm. However, men were more likely to be threatened with a firearm than women (p = 0.002), and this difference persisted by place of occurrence (p = 0.011).

“Fig 1. Cumulative Traumas by Sex” illustrates the cumulative number of traumas experienced by male and female participants along with the percentage of each sex in the sample. The percentages on the y-axis refer to percentages of men or women in the sample. Only 1.2% of females (1 participant) and no males endorsed that they had no trauma exposures. Almost 15% of the female participants experienced between 1 and 5 traumas compared to 11% of the male sample. Nearly 27% of female participants experienced between 6 and 10 different traumas compared to 20% of males. However, the percent of males becomes greater than the percent of females experiencing 11 to 15 types of traumas or more than 20 types of traumas.

## Discussion

Our study focuses on the timing and circumstances of trauma exposure of immigrants prior to their arrival to the U.S. and the degree to which exposures were related to sex. Prior work introduces the theory of “trilateral migration trauma” [30] among forced migrant children and families to understand mental health consequences; the framework refers to the potential traumas experienced in three phases: departure, migration, and relocation. The specificity in the timing and location of trauma experiences in our study aligns with the framework in [30] and provides an in-depth perspective of the experiences that may shape decisions to leave the home country versus experiences that happen along the journey to the U.S. Forced migration differs from traditional push-pull factors (e.g. economic opportunity, family reunification) because forced migrants may not always have the option of weighing traditional push-pull factors in their decisions to migrate [30]. Our study is consistent with theory from [30] because many of the immigrants in our sample left their home countries abruptly and under dangerous circumstances because of violence or threats of violence.

Our results reflect that immigrants traverse political instability [24, 31], lack of trust in institutions and impunity [32], and are increasingly vulnerable to being treated as commodities throughout their migration journey [33]. Conflict/war-like situations and experiencing the murders of family are consistent with previous studies about factors driving migration decisions for people seeking safety in the U.S [20, 34]. Our sample shows high levels of experiences of war-like conditions (e.g. 53% were exposed to frequent and unrelenting gun fire and 50% have been personally threatened with firearms) and experienced murders of family members and friends (52% of the sample). As a result of these war-like exposures, it is not unreasonable to think that migrants would seek to migrate away from such violence in the region. For example, prior work [34] demonstrates that past year victimization of self or a family member increases considering family migration to the U.S. by 30% among 49,000 survey respondents across 17 Latin American countries. Our sample is majority female of whom 18.8% are pregnant, and 94% of participants in our study were traveling with children. These two data points shed light on the changing demographics of migrants who are increasingly forced to migrate from their countries in search of safety and the prospect of a better future for their families. As our data shows, the journey to the U.S. is extremely dangerous, to say the least, for people such as pregnant women and children to undertake, unless they face few other options but to do so.

A majority of both men and women migrants in our study are fleeing and seeking safety rather than migrating for economic reasons (52.2% of respondents identified forced displacement as the main reason for migration). This trend is consistent with data from the United Nations High Commissioner for Refugees (UNHCR) which documents that worldwide more than two in five new asylum applications in 2022 were made by people from Latin America and the Caribbean [35]. Migration decisions often involve a complex set of factors (e.g. violence, lack of economic or educational opportunities, state level persecution or corruption) [30]. Asylum seekers in the U.S. experienced high levels of trauma in their home countries, perpetrated by gangs and state level actors, and also experience the denial of protections from the state when solicited, casting doubt on the safety of individuals who flee their countries in the case of involuntary return or deportation back to Latin American countries [20].

In general men and women experience an equally long list of traumas with two important exceptions. Women experience more sexual violence and rape, and men are exposed to combat-like situations [36] and are more likely to be threatened with firearms. This study sheds light on the extensive availability of firearms to the region [5, 37, 38], causing widespread fear, injury, and death. The rates of exposure to firearm violence in our study are alarmingly high compared to the U.S. adult population. Our participants are more than twice as likely to experience firearm threats compared to all U.S. adults, and twice as likely compared to Black U.S. adults, who experience the highest rates of firearm threats in the U.S. [39].

The ugliness of the migration experience does not discriminate between men and women, however, women disproportionately experience sexual violence. Prior work documents the high levels of sexual trauma perpetrated by gangs particularly in the Northern Triangle countries (Honduras, El Salvador and Guatemala) [20]. While sexual trauma has been documented during migration for many women in previous studies [19, 40], our findings point to significantly higher rates among female migrants (compared to male migrants) in both their home countries and during migration.

Our findings on the rates of rape and sexual victimization are similar to the rates experienced by women in the U.S. [41]. However, prior studies also document that there is a high prevalence of sexual and domestic violence against Latinx immigrant women in the U.S. and a low prevalence of help seeking due to immigration status and other factors [42, 43]. These studies raise a troubling prospect for the future of Latinx women in our sample, many of whom have already experienced high rates of violence, but perhaps whose agency for seeking help may be diminished in the U.S.

The prevalence of traumas experienced in this sample is enormous. Our study demonstrates the intersectional vulnerability of this population, which is shaped by socio-political, economic and gender disparities experienced in the home country and throughout the journey, and the prospect of facing less availability to help seeking and services once they are in the U.S. Our study reveals that individuals completing a migration journey from Latin America are traumatized and would benefit from targeted support and interventions that take the sex of the immigrant into consideration.

### Limitations

There are several limitations to this work. First, the study sample primarily consisted of individuals who came to the U.S. seeking asylum or other forms of protection. As a result, the participants had a high likelihood of experiencing trauma, and potentially reflected in our findings. However, a persistent challenge in conducting research with recent immigrants is the ability to access populations of recent immigrants to the U.S. It is challenging to access migrants who attempted but were unable to cross into the U.S., or the small proportion of immigrants who remain undetected by border security and other authorities once they are in the U.S. The current study focuses on migrants whom we can locate and access. While it is possible that trauma experiences may differ, studies of these other immigrant populations (e.g., did not cross into the U.S., or remain undetected after crossing into the U.S.) are generally unfeasible.

## Conclusion

This study is among the first to examine exposure to trauma differences by sex and place of occurrence in a population of recently arrived adult immigrants from Latin America. This study draws from a broader set of countries than prior studies. This study has important implications for clinical practice and research as it differentiates timing of traumatic events among male and female adults from Latin America. Interventions focused on reducing symptoms of anxiety, depression and PTSD that are part of a multi-modal model of care could prove beneficial for this population.

## Supporting information

S1 Checklist *PLOS ONE* clinical studies checklist. (DOCX)

## References

[1] Hamilton ER. Gendered disparities in Mexico-U.S. migration by class, ethnicity, and geography. Demogr Res. 2015;32:533–42.

[2] Colby SL, Ortman JM. Projections of the Size and Composition of the U.S. Population: 2014 to 2060. Curr Popul Rep US Census Bur. 2017;25–1143.

[3] Swanson K, Torres RM. Child Migration and Transnationalized Violence in Central and North America. J Lat Am Geogr. 2016 Nov 30;15(3):23–48.

[4] United Nations High Commissioner forRefugees. Children on the Run—Full Report [Internet]. [cited 2019 Oct 3]. Available from: https://www.unhcr.org/en-us/about-us/background/56fc266f4/children-on-the-run-full-report.html

[5] Muggah R, Aguirre Tobón K. Citizen Security in Latin America: Facts and Figures [Internet]. 2018 Apr [cited 2019 Oct 7]. Report No.: 33. Available from: https://igarape.org.br/en/citizen-security-in-latin-america-facts-and-figures/

[6] United Nations High Commissioner forRefugees. Global Trends—Forced Displacement in 2018—UNHCR [Internet]. 2019 [cited 2020 Feb 4]. Available from: https://www.unhcr.org/globaltrends2018/

[7] Eckhardt A, Zambrano EA, Nilsson D. Traumatic experiences and dissociation in a non-clinical group of university students in Ecuador: a cross-sectional study. J Child Adolesc Ment Health. 2018 Sep 2;30(3):191–202. doi: 10.2989/17280583.2018.1553780 30739606

[8] Grelotti DJ, Gerbasi ME, Eustache E, Fils-Aimé JR, Thérosmé T, Severe J, et al. Prevalence of stressful life events and their association with post-traumatic stress disorder among youth attending secondary school in Haiti. Psychiatry Res. 2018 Nov 1;269:369–75. doi: 10.1016/j.psychres.2018.08.074 30173043 PMC7050197

[9] Kappel RH, Livingston MD, Patel SN, Villaveces A, Massetti GM. Prevalence of Adverse Childhood Experiences (ACEs) and associated health risks and risk behaviors among young women and men in Honduras. Child Abuse Negl. 2021 May 1;115:104993. doi: 10.1016/j.chiabu.2021.104993 33611130 PMC8136622

[10] Mendoza-Mojica SA, Márquez-Mendoza O, Veytia-López M, Ramos-Lira LE, Orozco-Zavala R. Eventos potencialmente traumáticos y sintomatología postraumática en estudiantes de preparatoria. Salud Pública México. 2017 Oct 17;59(6, nov-dic):665–74. 10.21149/8716 29451636

[11] Orozco R, Borges G, Benjet C, Medina-Mora ME, López-Carrillo L. Traumatic life events and posttraumatic stress disorder among Mexican adolescents: results from a survey. Salud Pública México. 2008;50:s29–37. doi: 10.1590/s0036-36342008000700006 18373005

[12] ReliefWeb, Defense KKIN of. https://reliefweb.int/sites/reliefweb.int/files/resources/Neither-Security-nor-Justice_SGBV-Gang-Report-FINAL_0.pdf. 2018 [cited 2020 Oct 21]. El Salvador: Neither Security nor Justice: Sexual and gender-based violence and gang violence in El Salvador, Honduras, and Guatemala. Available from: https://www.ecoi.net/en/document/1436708.html

[13] Vallejo-Medina P, Pineda-Marín C, Soler F, Saaverdra-Roa A, Ortega LA. Validation of the Early Trauma Inventory Self Report-Short Form and trauma prevalence in Colombia. Psychol Trauma Theory Res Pract Policy. 2021;13:555–64. doi: 10.1037/tra0001008 33570988

[14] Stewart RW, Villalobos BT, Dueweke AR, Rodriguez JH, Nicasio AV, Alto M, et al. A Pilot Trial of Universal School-Based Mental Health Screening in El Salvador: Traumatic Stress in an Underresourced School Environment. J Trauma Stress. 2021;34(5):955–66. doi: 10.1002/jts.22716 34297864 PMC10019777

[15] Zlotnick C, Johnson J, Kohn R, Vicente B, Rioseco P, Saldivia S. Epidemiology of trauma, post-traumatic stress disorder (PTSD) and co-morbid disorders in Chile. Psychol Med. 2006 Nov;36(11):1523–33. doi: 10.1017/S0033291706008282 16854253

[16] Keller A, Joscelyne A, Granski M, Rosenfeld B. Pre-Migration Trauma Exposure and Mental Health Functioning among Central American Migrants Arriving at the US Border. PLOS ONE. 2017 Jan 10;12(1):e0168692. doi: 10.1371/journal.pone.0168692 28072836 PMC5224987

[17] Asnaani A, Hall-Clark B. Recent developments in understanding ethnocultural and race differences in trauma exposure and PTSD. Curr Opin Psychol. 2017 Apr 1;14:96–101. doi: 10.1016/j.copsyc.2016.12.005 28813327

[18] Tager AG, Aguilar Umaña I, Gereda M, Escobar Sarti C, Ramírez JM, Castro M. Violentas y violentadas: relaciones de género en las maras Salvatrucha y Barrio 18 del triángulo norte de Centroamérica. 2013 [cited 2020 Oct 21]; Available from: https://idl-bnc-idrc.dspacedirect.org/handle/10625/50910

[19] Kaltman S, Mendoza AH de Gonzales FA, Serrano A, Guarnaccia PJ. Contextualizing the trauma experience of women immigrants from Central America, South America, and Mexico. J Trauma Stress. 2011;24(6):635–42. doi: 10.1002/jts.20698 22144133 PMC3544003

[20] Cuneo CN, Huselton KE, Praschan NC, Saadi A, Gartland MG. What Counts As ‘Safe?’: Exposure To Trauma And Violence Among Asylum Seekers From The Northern Triangle. Health Aff (Millwood). 2021 Jul;40(7):1135–44. doi: 10.1377/hlthaff.2021.00082 34228513

[21] Cano M, Takeuchi DT. Recent/Childhood Adversities and Mental Disorders Among US Immigrants. Front Psychiatry [Internet]. 2020 [cited 2022 Nov 18];11. Available from: https://www.frontiersin.org/articles/10.3389/fpsyt.2020.573410 33312137 10.3389/fpsyt.2020.573410 PMC7703683

[22] Kremer P, Ulibarri M, Ferraiolo N, Pinedo M, Vargas-Ojeda AC, Burgos JL, et al. Association of Adverse Childhood Experiences with Depression in Latino Migrants Residing in Tijuana, Mexico. Perm J. 2019 Mar;23(1):18–031. 10.7812/TPP/18-031 PMC6307546 30589403

[23] Barton A, Novoa O, Sanchez M, Romano E, Cano M. Impact of pre-migration assets and pre-migration trauma on acculturative stress among recent Latinx immigrant young adults. Ethn Health. 2022 Aug 18;27(6):1410–27. doi: 10.1080/13557858.2021.1881766 33550826 PMC10616781

[24] Vazquez V, Rojas P, Cano MÁ, De La Rosa M, Romano E, Sánchez M. Depressive symptoms among recent Latinx immigrants in South Florida: The role of premigration trauma and stress, postimmigration stress, and gender. J Trauma Stress. 2022;35(2):533–45. doi: 10.1002/jts.22768 34973038 PMC9035034

[25] Mollica RF, Caspi-Yavin Y, Bollini P, Truong T, Tor S, Lavelle J. The Harvard Trauma Questionnaire: Validating a cross-cultural instrument for measuring torture, trauma, and posttraumatic stress disorder in Indochinese refugees. J Nerv Ment Dis. 1992;180(2):111–6. 1737972

[26] Shoeb M, Weinstein H, Mollica R. The Harvard Trauma Questionnaire: Adapting a Cross-Cultural Instrument for Measuring Torture, Trauma and Posttraumatic Stress Disorder in Iraqi Refugees. Int J Soc Psychiatry. 2007 Sep 1;53(5):447–63. doi: 10.1177/0020764007078362 18018666

[27] Berthold SM, Mollica RF, Silove D, Tay AK, Lavelle J, Lindert J. The HTQ-5: revision of the Harvard Trauma Questionnaire for measuring torture, trauma and DSM-5 PTSD symptoms in refugee populations. Eur J Public Health. 2019 Jun 1;29(3):468–74. doi: 10.1093/eurpub/cky256 30561573

[28] Alegria M, Takeuchi D, Canino G, Duan N, Shrout P, Meng XL, et al. Considering Context, Place, and Culture: The National Latino and Asian American Study. Int J Methods Psychiatr Res. 2004;13(4):208–20. doi: 10.1002/mpr.178 15719529 PMC2774128

[29] Kessler RC, Ustün TB. The World Mental Health (WMH) Survey Initiative Version of the World Health Organization (WHO) Composite International Diagnostic Interview (CIDI). Int J Methods Psychiatr Res. 2004;13(2):93–121. doi: 10.1002/mpr.168 15297906 PMC6878592

[30] Clauss-Ehlers CS. Forced Migration among Latinx Children and their Families: Introducing Trilateral Migration Trauma as a Concept to Reflect a Forced Migratory Experience. J Infant Child Adolesc Psychother. 2019 Oct 2;18(4):330–42.

[31] Abaya M, Lesley B, Williams C, Chaves-Gnecco D, Flores G. Forcible Displacement, Migration, and Violence Against Children and Families in Latin America. Pediatr Clin North Am. 2021 Apr;68(2):371–87. doi: 10.1016/j.pcl.2020.12.003 33678292

[32] Walsh SD, Menjívar C. Impunity and multisided violence in the lives of Latin American women: El Salvador in comparative perspective. Curr Sociol. 2016 Jul 1;64(4):586–602.

[33] Vogt WA. Crossing Mexico: Structural violence and the commodification of undocumented Central American migrants. Am Ethnol. 2013;40(4):764–80.

[34] Wood CH, Gibson CL, Ribeiro L, Hamsho-Diaz P. Crime Victimization in Latin America and Intentions to Migrate to the United States. Int Migr Rev. 2010 Mar 1;44(1):3–24.

[35] UNHCR [Internet]. [cited 2023 Sep 8]. Global Trends Report 2022. Available from: https://www.unhcr.org/global-trends-report-2022

[36] Tolin DF, Foa EB. Sex differences in trauma and posttraumatic stress disorder: A quantitative review of 25 years of research. Psychol Trauma Theory Res Pract Policy. 2008;S:37–85. 10.1037/0033-2909.132.6.959 17073529

[37] Briceño-León R, Villaveces A, Concha-Eastman A. Understanding the uneven distribution of the incidence of homicide in Latin America. Int J Epidemiol. 2008 Aug 1;37(4):751–7. doi: 10.1093/ije/dyn153 18653511

[38] Witkowski TH. Provisioning Firearms in Latin America: Historical Development and Societal Consequences. J Macromarketing. 2022 Jun 1;42(2):169–90.

[39] Schumacher S, Presiado M, Valdes I, 2023. Americans’ Experiences With Gun-Related Violence, Injuries, And Deaths [Internet]. KFF. 2023 [cited 2023 Apr 28]. Available from: https://www.kff.org/other/poll-finding/americans-experiences-with-gun-related-violence-injuries-and-deaths/

[40] Infante C, Idrovo AJ, Sánchez-Domínguez MS, Vinhas S, González-Vázquez T. Violence Committed Against Migrants in Transit: Experiences on the Northern Mexican Border. J Immigr Minor Health. 2012 Jun 1;14(3):449–59. doi: 10.1007/s10903-011-9489-y 21655940

[41] Smith SG, Zhang X, Basile KC, Merrick MT, Wang J, Kresnow M jo, et al. The National Intimate Partner and Sexual Violence Survey (NISVS): 2015 Data Brief–Updated Release. Atlanta, GA: National Center for Injury Prevention and Control, Centers for Disease Control and Prevention.; 2018 Nov. (The National Intimate Partner and Sexual Violence Survey).

[42] Zadnik E, Sabina C, Cuevas CA. Violence Against Latinas: The Effects of Undocumented Status on Rates of Victimization and Help-Seeking. J Interpers Violence. 2016 Mar 1;31(6):1141–53. doi: 10.1177/0886260514564062 25540190

[43] Hass GA, Dutton MA, Orloff LE. Lifetime Prevalence of Violence Against Latina Immigrants: Legal and Policy Implications. Int Rev Vict. 2000 Jan 1; 7(1–3):93–113.

