# Supplementary material for: Traumatic experiences and place of occurrence: An analysis of sex differences among a sample of recently arrived immigrant adults from Latin America
Source: PLoS One. 2024 Jun 14;19(6):e0302363. doi: 10.1371/journal.pone.0302363 (PMC11178180; doi:10.1371/journal.pone.0302363)
Supplement: Attachment — Submitted filename: PlosOne_Response_Letter_FINAL.docx [file pone.0302363.s002.docx]

Dear Editor and Reviewers,

Thank you to the Editor for their helpful observations. Overall, we have made modifications to the manuscript based on the Editor’s and Reviewers’ comments. We address the comments of Reviewers below. We believe the suggestions we received have made this manuscript better and we have tried to be responsive to your helpful suggestions.

- In terms of making the dataset publicly available, we do not have explicit consent from our participants to make our data publicly available. In fact, because of the sensitive nature of this data and the vulnerability of this population, we have told our participants that this data is only available in a de-identified way to members of the research team. We realize that we are in an era of open data, but we also know of the reluctance of certain understudied and extremely important populations to participate in research. Part of our ability to collect this data is the trust that participants place in us. We value their trust greatly and it is vital to respect our commitment to the trust placed in us.
- However, as requested by the journal, we provide a non-author point of contact where data requests can be sent.

We thank Reviewer #1 for their helpful comments and insights. Based on the feedback we received we have:

- Edited the discussion section to reflect a more in-depth analysis of our results, by placing it within existing theory and evidence in the field.
- We appreciate the observation that we were repeating some of our results, and we have eliminated repeating of findings the discussion and instead include a more in-depth analysis of what our results may mean for the current state of the literature.
- We have revised some of the conclusions that are considered premature and provided an overall better context for our discussion section.

Thank you to Reviewer #2 for their helpful suggestions and comments. In response to them we have made the following changes:

- We moved the paragraphs in the introduction according to the Reviewer’s suggestions.
- We have clarified the responses for the question on the reason for migration. We clarify that actually, rather than an open-ended answer (we described it incorrectly in our previous draft), the reason for migration is a multiple choice response and we clarify what the possible answers to this question are in the methods section of the manuscript.
- We modified the language to reflect percentages as numbers

We thank you again for considering this manuscript for peer-review in your Journal. Please let us know if there is anything else you may need.

Sincerely,

Laura Vargas, PhD, LMSW, MPA (corresponding author)
