# Supplementary material for: Traumatic experiences and place of occurrence: An analysis of sex differences among a sample of recently arrived immigrant adults from Latin America
Source: PLoS One. 2024 Jun 14;19(6):e0302363. doi: 10.1371/journal.pone.0302363 (PMC11178180; doi:10.1371/journal.pone.0302363)
Supplement: Attachment — Submitted filename: PlosOne_Response_Letter_030524.docx [file pone.0302363.s003.docx]

Dear Editor and Reviewers,

Thank you to the Editor for their helpful observations and comments. We appreciate the opportunity to revise this manuscript and resubmit it for consideration in PlosOne.

To our knowledge, we do not know of any references cited in our manuscript that have been retracted from publication.

We thank Reviewer #1 once again for their helpful comments and insights. To address the reviewer’s comments we made the following changes to our submission:

- We thank the reviewer for their comment on explicitly stating the rationale for our analysis. We add a sentence at the end of our Introduction section doing so, which we hope will be helpful.
- We thank the reviewers for highlighting the importance of the proportion of our sample who are pregnant or traveling with a pregnant partner. We make reference to this portion of our sample in more detail in the result page 6 lines 179-181, and again in our discussion of results in page 13 lines 261-266.
- We thank the reviewer for their comment regarding the richness of our discussion. WE have tried to incorporate some of the suggestions of reviewers in the previous round of comments and in this round of comments in the hopes that we can improve this manuscript. We also try to balance the need for our manuscript to be clear, concise, and precise in its content to meet the journal’s very reasonable requirements.
- We clarify in the measures section of our manuscript what is meant by the “previous year” page 5, lines 140-141. Because the survey questions is meant to ask about employment in the 12 months prior to the date of interviews, we do not refer to a specific year of employment because our interviews were conducted over the course of several weeks/months the year in reference may be different. In addition, the way we phrase the question is “how many months did you work in the previous 12 months?” – an explanation that we include in our manuscript.
- We included two clarifying sentences in page 13, lines 256-260 about how war-like conditions in the Latin American region may be linked to migrants’ decisions to leave their home countries.
- We have made the proper adjustments for references to appear in sequential order.

We thank you again for considering this manuscript for peer-review in your Journal. Please let us know if there is anything else you may need.

Sincerely,

Laura Vargas, PhD, LMSW, MPA (corresponding author)
